# Proximal and Distal Regions of Pathogenic Th17 Related Chromatin Loci Are Sequentially Accessible During Pathogenicity of Th17

**DOI:** 10.3389/fimmu.2022.864314

**Published:** 2022-04-19

**Authors:** Luni Hu, Xingyu Zhao, Peng Li, Yanyu Zeng, Yime Zhang, Yang Shen, Yukai Wang, Xiaolin Sun, Binbin Lai, Chao Zhong

**Affiliations:** ^1^ Beijing Key Laboratory of Tumor Systems Biology, Institute of Systems Biomedicine, School of Basic Medical Sciences, Peking University Health Science Center, Beijing, China; ^2^ School of Basic Medical Sciences, Peking University Health Science Center, Beijing, China; ^3^ Department of Rheumatology and Immunology, Peking University People’s Hospital, Beijing, China; ^4^ Beijing Key Laboratory for Rheumatism Mechanism and Immune Diagnosis (BZ0135), Peking University People's Hospital, Beijing, China; ^5^ Biomedical Engineering Department, Peking University, Beijing, China; ^6^ Institute of Medical Technology, Peking University Health Science Center, Beijing, China; ^7^ Department of Dermatology and Venereology, Peking University First Hospital, Beijing, China; ^8^ National Health Commission (NHC) Key Laboratory of Medical Immunology, Peking University, Beijing, China; ^9^ Key Laboratory of Molecular Immunology, Chinese Academy of Medical Sciences, Beijing, China

**Keywords:** autoimmune disease, regulatory Th17, pathogenic Th17, pro-inflammatory Th1, chromatin accessibility, transcriptional regulation

## Abstract

Pathogenic Th17, featured by their production of pro-inflammatory cytokines, are considered as a key player in most autoimmune diseases. The transcriptome of them is obviously distinct from that of conventional regulatory Th17. However, chromatin accessibility of the two Th17 groups have not been comprehensively compared yet. Here, we found that their chromatin-accessible regions(ChARs) significantly correlated with the expression of related genes, indicating that they might engage in the regulation of these genes. Indeed, pathogenic Th17 specific ChARs (patho-ChARs) exhibited a significant distribution preference in TSS-proximal region. We further filtered the patho-ChARs based on their conservation among mammalians or their concordance with the expression of their related genes. In either situation, the filtered patho-ChARs also showed a preference for TSS-proximal region. Enrichment of expression concordant patho-ChARs related genes suggested that they might involve in the pathogenicity of Th17. Thus, we also examined all ChARs of patho-ChARs related genes, and defined an opening ChAR set according to their changes in the Th17 to Th1 conversion. Interestingly, these opening ChARs displayed a sequential accessibility change from TSS-proximal region to TSS-distal region. Meanwhile, a group of patho-TFs (transcription factors) were identified based on the appearance of their binding motifs in the opening ChARs. Consistently, some of them also displayed a similar preference for binding the TSS-proximal region. Single-cell transcriptome analysis further confirmed that these patho-TFs were involved in the generation of pathogenic Th17. Therefore, our results shed light on a new regulatory mechanism underlying the generation of pathogenic Th17, which is worth to be considered for autoimmune disease therapy.

## Introduction

Th17 cells provide crucial protections against extracellular bacteria and fungi (1). However, they also participate in pathogenesis of many autoimmune diseases, such as multiple sclerosis (MS), rheumatoid arthritis (RA), systemic lupus erythematosus (SLE), and psoriasis. For example, MS is a progressive autoimmune disease affecting central nervous system (2). Myelin oligodendrocyte glycoprotein (MOG), a self-antigen at the outer surface of myelin sheath, ignites auto immune responses of T cells and B cells (3). Particularly, skewed T cell differentiation towards pathogenic Th17 is frequently found to correlate with the disease development (3, 4). The role of these Th17 cells is obviously distinct from those generated during infections. In both conditions, Th17 all express effector cytokine IL-17, as well as their master transcriptional regulator, retinoid-related orphan nuclear receptor gamma t (RORγt). Nevertheless, recent studies have also revealed a significant difference between them (5). Thus, according to the functional and transcriptional discrepancies, the Th17 responding during infections are usually termed as protective or regulatory Th17, whereas the other Th17 exhibiting pro-inflammatory features during autoimmune diseases are often termed as pathogenic or pro-inflammatory Th17 (6). The difference between their generation is still unclear. Unravelling the underlying mechanisms could provide new therapeutic strategies for many autoimmune diseases. Thus, related studies have attracted many attentions in the past (7, 8).

Several animal models and *in vitro* cell culture systems have been developed to interpret the difference between the two Th17 subgroups. Experimental autoimmune encephalitis (EAE) is frequently used as a mice model of MS. The autoimmune response in the mice is induced by the MOG peptide (9–29). Similar as in MS patients, MOG-specific pathogenic Th17 dramatically expand in mouse spinal cord, which drives the disease progression (5, 30). On the other hand, the regulatory Th17 subgroup is usually found to be enriched in gut at steady state, or, can be stimulated to expand by certain gut microbes, such as segmented filamentous bacteria (SFB) (31). They play crucial roles in maintaining intestinal homeostasis and protecting host from infectious pathogens (1). Gene expression differences between these pathogenic Th17 generated during EAE and regulatory Th17 residing in gut are often analyzed to reveal key regulators involved in their generation (32). In addition, to better interpret the roles of key regulators in the generation of the two Th17 subgroups, *in vitro* conditions for their differentiation have also been established. IL-23 and IL-1β are critical in the pathogenesis of EAE (33, 34). In line with it, IL-23 and IL-1β, together with IL-6, can dictate an *in vitro* differentiation of the pathogenic Th17 from naïve T cells. The pathogenicity of these cells is confirmed by their similar gene expression profile as the Th17 found in EAE, as well as their capacity to induce EAE after adoptively transferred into mice (35). While regulatory Th17 can be induced *in vitro* by TGF-β and IL-6, and they are non-pathogenic to mice after adoptive transfer (35). These *in vitro* differentiated Th17 make it more convenient to reveal the regulatory mechanisms underlying their generation. Together, with these pathogenic and regulatory Th17 generated *in vivo* or *in vitro*, it is possible to intensively interpret the differences between the two Th17 subgroups.

Pathogenic Th17 exhibit an obvious different gene expression profile in comparison to regulatory Th17. Genes specifically expressed by regulatory Th17 include *Maf*, *Ahr*, *Ikzf3*, *Il10*, *Il9*, and *Il21* (36–41), while pathogenic Th17-specific genes contain *Ctla4*, *Gpr65*, *Plzp*, *Cd5l*, *Ccr6*, *Tbx21*, *Id3*, *Ccl20*, *Ccl5*, *Ifng*, *Csf2*, and *Tnf* (5, 32, 42–45). Among them, most are demonstrated to play critical roles in the generation or function of these two Th17 subgroups. The effector genes in pathogenic Th17 are related to their pathogenicity in EAE. They include *Ifng*, *Tnf*, *Csf2*, *Ccl20*, and *Ccl5*, which are much similar to the effector genes of pro-inflammatory Th1 cells (5, 46–49). Their blockade can efficiently ameliorate the autoimmune responses (50–52). In addition, most transcriptional regulators of these effector cytokines, such as *Tbx21* and *Id3* (44, 45), are also key regulators in Th1, which further explains why pathogenic Th17 usually exhibit Th1-like features (53).

A conversion of the pathogenic Th17 to pro-inflammatory Th1 is also frequently found during EAE and other autoimmune diseases (53, 54). Many studies suggest that this conversion is crucial in the disease progression (53, 54). The precise mechanism promoting the conversion is still elusive. IL-23, belonging to IL-12 cytokine family, is a key driver of pathogenic Th17 (54, 55). It is also found to correlate with the initiation of T-bet expression (44). IL-23 shares a common p40 subunit with IL-12, an upstream cytokine essential for Th1 differentiation. Therefore, IL-23, like IL-12, can activate STAT4 and thus induce the expression of T-bet, which is critical in the acquisition of Th1-like features by pathogenic Th17. In feedback, the increased T-bet can directly bind to *Il23r* locus to promote its transcription, which finally promotes the T-bet expression (9). Regulatory roles of T-bet and RORγt are usually contrary. As a result, pathogenic Th17 will gradually enhance their T-bet expression and attenuate the RORγt expression, ending with a complete conversion to pro-inflammatory Th1. However, in contrast to the *bona fide* Th1 cells that directly differentiate from naïve CD4^+^ T cells, these ‘ex-Th17’ Th1 still maintain their IL-23R expression (10). Whether they still have other differences is unclear. And, other regulators should also participate in the pathogenic Th17 to pro-inflammatory Th1 conversion. Thus, further studies are required to comprehensively understand this process.

Chromatin accessibility is critical in regulating gene expression in mammalian cells (11). When genomic DNA is tightly wrapped in nucleosomes, transcription factor binding is generally prevented, which results in attenuated expression of related genes. Epigenetic modifications on histone and chromatin dynamically regulate the accessibility of genomic DNAs, leading to downstream gene expression changes. Several techniques have been developed to recognize chromatin accessible regions (ChARs). In general, they are all based on accessibility of chromatin DNA to enzymes, such as Tn5 transposase, DNAse I, and MNase (11, 12). These technologies are further combined with deep sequencing to get the chromatin landscape (12). Currently, assay for transposase-accessible chromatin with high throughput sequencing (ATAC-seq) is the most frequently used technique for chromatin accessibility studies. ChARs are permissive for transcriptional regulation. Thus, further analysis of transcription factor binding motifs within them can help to predict the underlying regulatory mechanisms. According to the frequency of transcription factor binding motifs within ChARs, it enables the prediction of key transcription factors in gene regulation (13). Therefore, the ATAC-seq based chromatin accessibility analysis is a powerful tool for unraveling the regulatory mechanisms of gene expression.

The differences of gene expression between pathogenic and regulatory Th17, associated with their regulations and functions, have attracted many attentions in the past (32). However, the difference in their DNA accessibility is still lack of intensive studies. The DNA accessibility is usually of major importance in regulating gene expression. Thus, its difference between the two Th17 subgroups is also worth exploring. Especially, further understanding the regulatory mechanisms underlying pathogenic Th17 generation may provide new clinical strategies for related autoimmune diseases.

## Methods

### Data Acquisition

Raw gene expression profiles (RNA-Seq) and chromatin accessibility profiles (ATAC-Seq) of ileum and CNS-infiltrated Th17 cells were retrieved from Sequence Read Archive (SRA) under accession number SRP187402. Raw gene expression profiles (RNA-Seq) and chromatin accessibility profiles (ATAC-Seq) of Th1 cells were retrieved from Sequence Read Archive (SRA) under accession number SRP221527. Single-cell gene expression profiles of Th17 cells in CNS from EAE mice were retrieved from Sequence Read Archive (SRA) under accession numbers SRP344569.

### Bulk RNA-Seq Data Processing

RNA-Seq reads were extracted into fastq files using fasterq-dump (v 2.11.0) and mapped to mm10 using HISAT2 (v 2.2.1). Gene expression level was counted by featureCounts (v 2.0.3) against mouse GRCm38 genome assembly (v 102). Transcripts-per-million (TPM) values were calculated with R package scuttle (v 1.0.4). Briefly, truly expressed genes (TPM > 5 in both repeats of either group) were used for downstream analyses.

### ATAC-Seq Data Processing

ATAC-Seq reads were extracted into fastq files using fasterq-dump (v 2.11.0). Quality trimming and primer removal from the raw fastq files were performed with Trimmomatic (v 0.36), using the following parameters: LEADING:15 TRAILING:15 SLIDINGWINDOW:4:15 MINLEN:36. The trimmed reads were aligned to mm10 using Bowtie2 (v 2.4.4). The aligned reads were sorted using samtools (v 1.13), and duplicates were marked and removed using PICARD (v 0.7).

### Peak Universe Generation and Differential ChAR Determination

Peak-calling for ATAC-Seq was performed with MACS (v 2.2.7.1) on bam files, using a q-value threshold of 0.01. Consensus peak from all Th17 cells were merged to create a raw peak universe, and then, it was further filtered according to the truly expressed genes in Th17 to get the final peak universe of 16,548 regions. ATAC-Seq reads within each peak region was quantified using BEDtools (v 2.27.1) to generate a raw count matrix. Transcripts-per-million (TPM) values for peak-level counts were calculated with R package scuttle (v 1.0.4). For differential ChAR determination, a criterion of fold change > 2 was used.

### Peak Annotation, GO Enrichment, and Motif Enrichment

Annotation of genomic regions to their neighboring genes were operated by HOMER (v4.10). GO enrichment was performed by R package clusterProfiller (v 3.18.0), and hypergeometric test was used to measure the significance. Motif enrichment analyses were performed by MEME Suit (v4.11.2).

### Conservation Analysis

The evolutionary conservation score of each ChAR (in either patho-ChARs or reg-ChARs) was measured using bigWigAverageOverBed command, with phastCons conservation scores from mm10.60way.phastCons.bw (downloaded from UCSC), which contained conservation scores for alignments of 59 vertebrate genomes with mouse genome generated by phastCons program (14, 15). The conservation score cutoff for conserved ChARs or non-conserved ChARs was set at 0.5, referring to Methods by Hong Sun and Yu (16).

### Expression Concordance Analysis of ATAC-Seq and RNA-Seq

Concordant ChARs were defined as differential ChARs (fold change > 2) with their annotated genes also exhibiting gene expression concordance (fold change > 2). Whereas, unconcordant ChARs were defined as differential ChARs with irrelevant or contrary expression change of their annotated genes. The accuracy of concordant and unconcordant ChARs in distinguishing pathogenic Th17, regulatory Th17 and pro-inflammatory Th1 cells was further examined with Spearman correlation analysis.

### Single-Cell RNA-Seq Data Analysis

Single-cell RNA-Seq raw count matrix was processed with R package Seurat (v 4.0) to remove low quality cells and obtain the normalized gene expression. Single cell clustering was performed with shared nearest neighbors (SNN) algorithm, and dimension reduction was operated with Uniform Manifold Approximation and Projection (UMAP) algorithm. Pseudotime analysis was inferred with Monocle (v 2.18.0). Module scores for gene expression of single cell was calculated with AddModuleScore function in Seurat. Transcription regulation network based on expression correlation of TFs and target genes was constructed with Cytoscape (v 3.9.0).

### Statistics and Data Visualization

Two-group two-sided Mann-Whitney U tests were run to compare differences in the levels of mRNA expression of neighboring genes. Asterisks were used to indicate significance as follows: *P < 0.05, **P < 0.01, ***P < 0.001, and ****P < 0.0001 (**Figure S1B**). The statistical significance of GO enrichment and motif enrichment were calculated by two-sided hypergeometric test. P values less than 0.05 were considered as indicating a significant difference. ATAC-Seq tracks were visualized using Integrative Genomics Viewer (v 2.11.0). Heatmap visualization was performed by pheatmap (v 1.0.12). Dot plots, scatter plots and histograms were operated by ggplot2 (v 3.3.3).

## Results

### DNA Accessibility in Pathogenic and Regulatory Th17 Correlates With the Expression of Their Related Genes

To compare the DNA accessibility between pathogenic and regulatoryTh17, a set of ATAC-seq and RNA-seq results (GSE# 127768) for Th17 from spinal cord of mice with EAE and ilium of healthy mice were used (17). First, chromatin accessible regions (ChARs) were profiled from the ATAC-seq data, and those with more than 2fold changes in either of Th17 subgroups were defined as pathogenic Th17-specific ChARs or regulatory Th17-specific ChARs (patho-ChARs or reg-ChARs) (**Figure S1A**). Then, we wondered whether this difference in DNA accessibility correlated with their gene expression changes. All ChARs in pathogenic Th17 and regulatory Th17 were binned into six groups, based on their accessibility difference between the two Th17 subgroups. When integrating the RNA-seq results, we observed that, for ChARs with more than 2-fold changes, the expression of their related genes in average showed a significant and concordant change. For the residual comparable ChARs, their related genes had also showed equivalent expression (**Figure S1B**). Effector cytokine expression in pathogenic or regulatory Th17 was particularly important for their functions. *Ifng*, *Tnf*, and *Csf2* were prominently expressed by pathogenic Th17, while *Il17a*, *Il17f*, *Il10* and *Il21* were substantially expressed by regulatory Th17 (5, 48, 49). Consistently, obvious DNA accessibility differences at their gene loci were also identified, suggesting that these specific ChARs in the two Th17 subgroups might closely associate with their effector function (**Figure S1C**). Together, DNA accessibility difference between pathogenic and protective Th17 was consistent with their gene expression change. Moreover, they should also relate with the difference in their transcriptional regulations. Therefore, it was worthful to further compare the patho-ChARs and reg-ChARs.

### Patho-ChARs and Reg-ChARs Differ in Their Distribution at Gene Loci

**Figure 1:**
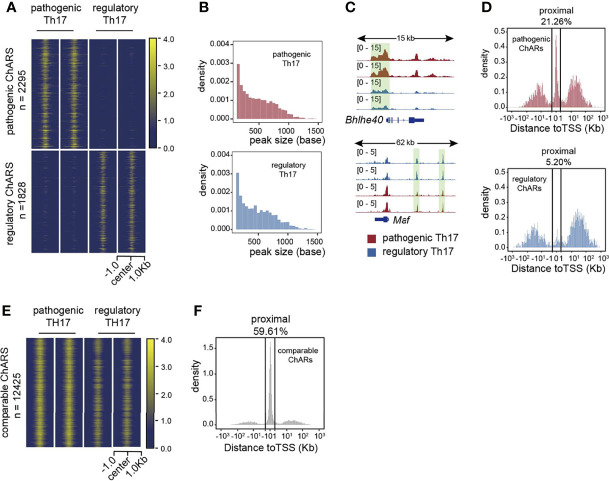
Distributions of patho-ChARs and reg-ChARs at gene loci are different. **(A)** Heatmap of chromatin accessibility difference between pathogenic and regulatory Th17. **(B)** Peak width distributions of patho-ChARs (red) and reg-ChARs (blue). **(C)** ATAC-Seq tracks at *Bhlhe40* and *Maf*gene loci in pathogenic and regulatory Th17. **(D)** Distance of patho-ChARs (red) and reg-ChARs (blue) to their neighboring TSSs. Percentage of ChARs found within +1 to -1 Kb of TSS were calculated. **(E)** Heatmap of chromatin accessibility of com-ChARs in pathogenic and regulatory Th17. **(F)** Distance of com-ChARs to their neighboring TSSs. Percentage of ChARs found within +1 to -1 Kb of TSS were calculated.

Based on the identification of patho-ChARs and reg-ChARs, next we wondered whether they reflected any regulatory difference between the two Th17 subgroups. The numbers of patho-ChARs and reg-ChARs (2295 and 1828, respectively) were almost identical (**Figure 1A**). Likewise, the peak widths between them were also similar (**Figure 1B**). However, when checking their distributions at gene loci, we did find some differences. Took a pathogenic Th17-specific gene *Bhlhe40* and a regulatory Th17-specific gene *Maf* as examples here (**Figure 1C**). The patho-ChAR for *Bhlhe40* was found to locate in proximate to its TSS. Whereas, the reg-ChAR for *Maf* located in a region distal to its TSS. To further confirm this difference, we profiled the distribution of all patho-ChARs and reg-ChARs around TSSs of their correlated genes. As expected, in a TSS-proximal region of -1 kb ~ +1 kb, the frequency of patho-ChARs was significantly higher (**Figure 1D**). In contrast, the reg-ChARs were mainly distributed in TSS-distal regions. TSS-proximal regions, containing promoters, were usually considered to be essential in initiating a prompt gene expression (18, 19). Whereas, TSS-distal regions, containing both enhancers and suppressors, could provide more delicate regulations (18, 19). Thus, these results suggested that, in regulatory Th17, majority of their specific genes had received additional regulations from the distal reg-ChARs, which might benefit the cells with a more precise regulation of their effector function. Though specific distal ChARs also existed in the pathogenic Th17, the enrichment of proximal patho-ChARs was more impressive. Considering the pro-inflammatory feature of pathogenic Th17, it might correlate with the unleashed effector gene expression.

Meanwhile, we also profiled the distribution of 12425 residual ChARs that were comparable between the two Th17 subgroups (termed as com-ChARs) (**Figure 1E**). They also showed an obvious preference to the TSS-proximal region (**Figure 1F**). Thus, the proximal regulation perhaps was generally used to ensure a concise but efficient expression of most genes in a cell. However, for those genes related with effector functions of immune cells, the TSS-distal regions turned to become more accessible, to enable additional transcriptional and epigenetic regulations. This change probably was required to finely organize their effector functions. While the appearance of TSS-proximal patho-ChARs, and their potential roles in initiating prompt regulations of effector genes, such as *Ifng*, *Tnf*, and *Csf2*, perhaps led to the development of pathogenic Th17.

### Conserved Patho-ChARs and Reg-ChARs Also Display Similar Distribution Patterns

Cis-regulatory elements (CREs), including promoters, enhancers, and suppressors, were critical in regulating gene expression (18, 19). Their sequence were often conserved among different species (20, 21). *Vice versa*, conserved ChARs were more likely to serve as functional CREs. Therefore, next we filtered the patho-ChARs and reg-ChARs by conservation, and further analyzed their distribution in gene loci. The conserved ChARs were defined based on their conservation scoring (> 0.5) (**Figures S2A, B**). Accordingly, we identified 199 conserved patho-ChARs and 157 conserved reg-ChARs. In TSS-distal regions, these conserved patho-ChARs and reg-ChARs only exhibited a mild difference in number (122 and 147) (**Figure S2C**). However, in TSS-proximal regions, their numbers were dramatically different (77 and 10) (**Figure S2C**), similar as what we had just found (**Figure 1D**). In addition, comparing to the proportion of TSS-proximal patho-ChARs we had just found (21.26%) (**Figure 1D**), in conserved patho-ChARs the proportion of TSS-proximal patho-ChARs increased to 38.69% (**Figures S2D, E**). Since the conserved ChARs were potentially functional (20, 21), this increment further suggested that the proximal regulation was critical in pathogenic Th17. In opposite, in conserved reg-ChARs, the proportion of TSS-proximal reg-ChARs (6.37%) did not change too much (**Figures S2D, E**).

As we mentioned, the conserved ChARs were more likely to be functional in gene expression regulation (20, 21). Thus, we wondered whether these conserved ChARs we identified here had exhibited any correlation with the expression of their related genes. So, an integrated analysis with the RNA-seq result was performed (**Figure S2F**). In TSS-proximal regions, we found that 38 conserved patho-ChARs (49.38%) and 5 conserved reg-ChARs (50%) positively correlated with the expression of their related genes, while 9 conserved patho-ChARs (11.69%) negatively correlated with the expression of their related genes (**Figure S2G**). However, there were also 30conserved patho-ChARs (38.96%) and 5 conserved reg-ChARs (50%) irrelevant with the expression of their related genes. In TSS-distal region, 27 conserved patho-ChARs (22.13%) and 49 conserved reg-ChARs (33.33%) positively correlated with their related genes, while 17 conserved patho-ChARs (13.93%) and 16 conserved reg-ChARs (10.88%) negatively correlated with their related genes (**Figure S2G**). Together, these results suggested that though most of the conserved ChARs showed regulatory functions, their correlation with gene expression was uncertain.

### Expression Concordant Patho-ChARs Exhibit Enriched Pro-Inflammatory Features and Associate With Th17 to Th1 Conversion

**Figure 2:**
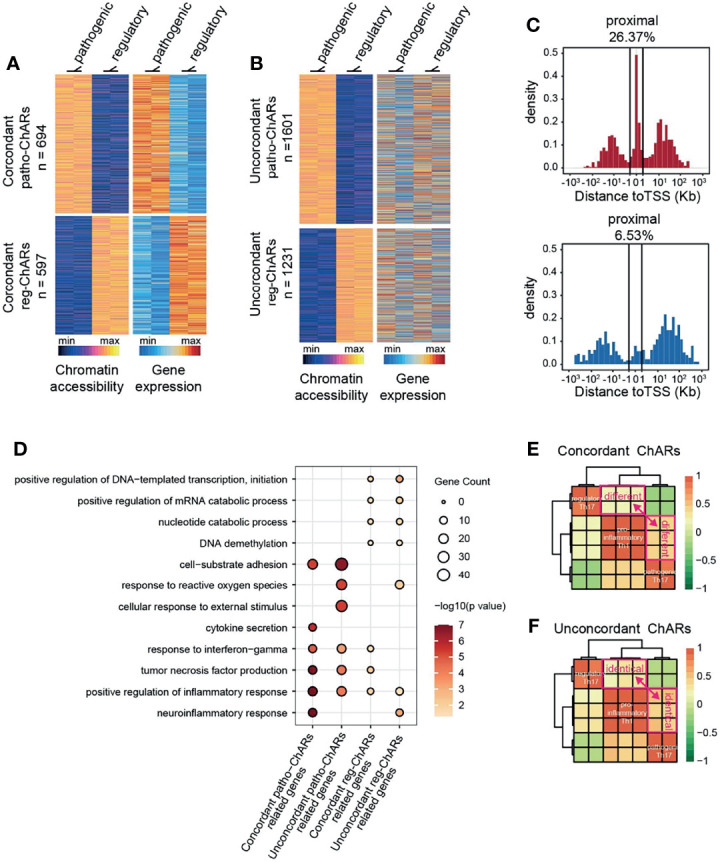
Expression concordant patho-ChARs associate with Th17 to Th1 conversion. **(A)** Heatmap of expression concordant ChARs in pathogenic and regulatory Th17 (left), and their related gene expression (right). **(B)** Heatmap of expression unconcordant ChARs (left) and their related gene expression (right). **(C)** Distance of concordant patho-ChARs and concordant reg-ChARs to their neighboring TSSs. Percentage of ChARs found within +1 to -1 Kb of TSS were calculated. **(D)** GO enrichment of concordant patho-ChARs, concordant reg-ChARs, unconcordant patho-ChARs, and unconcordant reg-ChARs. **(E)** Similarity between pathogenic Th17, regulatory Th17 and pro-inflammatory Th1 in accessibility to concordant patho-ChARs and reg-ChARs. **(F)** Similarity between pathogenic Th17, regulatory Th17 and pro-inflammatory Th1 in accessibility to unconcordant patho-ChARs and reg-ChARs.

Though the conserved ChARs tended to be functional in both Th17 subgroups, it was still difficult to predict the expression change of their related genes. Thus, next we directly analyzed the ChARs concordant with their related genes in pathogenic and regulatory Th17, to see whether any underlying regulatory mechanisms existed. Within Patho-ChARs or reg-ChARs, those positively correlated with the expression of their related genes were defined as concordant ChARs (**Figure 2A**), while the residual ChARs, irrelevant or negatively correlated with their related genes, were all defined as unconcordant ChARs (**Figure 2B**). 694 concordant patho-ChARs and 597 concordant reg-ChARs were identified, while 1601 patho-ChARs and 1231 reg-ChARs were classified as unconcordant ChARs. We speculated that concordant ChARs should be more enriched in TSS-proximal region, as promoters responsible for gene expression initiation were typical concordant ChARs. Indeed, in comparison to patho-ChARs or reg-ChARs, concordant patho-ChARs or reg-ChARs were more frequently appeared in TSS-proximal region (26.37%, 6.53%), while concordant patho-ChARs were still more enriched than concordant reg-ChARs there (**Figure 2C**).

Next, we explored whether these concordant ChARs, as well as their related genes, played crucial roles in both Th17 subgroups. Therefore, we applied the four ChAR subsets, (concordant patho-ChARs, unconcordant patho-ChARs, concordant reg-ChARs, and unconcordant reg-ChARs) to GO enrichment analysis (**Figure 2D**). In pathogenic Th17, the roles of concordant patho-ChARs and unconcordant patho-ChARs exhibited a dramatic difference. Concordant patho-ChARs were substantially enriched in pro-inflammatory features, such as neuroinflammation response, positive regulation of inflammation response, tumor necrosis factor production, response to interferon gamma and cytokine secretion. However, in unconcordant patho-ChARs, these features were not obviously enriched comparatively. In contrast, they were enriched in features of cellular response to external stimulus, response to reactive oxygen species and cell-substrate adhesion. On the other hand, in regulatory Th17, the difference between concordant reg-ChARs associated features and unconcordant reg-ChARs associated features was not that much remarkable. Therefore, the concordant patho-ChARs might be particularly important to the pathogenic feature of Th17.

According to the literature, a two-step Th17 to Th1 conversion existed during autoimmune diseases (53, 54). The regulatory Th17 would first switch to pathogenic Th17, followed with a final switch to pro-inflammatory Th1. The significant enrichment of concordant patho-ChARs in pro-inflammatory features raised a possibility that they might involve in regulating the Th17 to Th1 conversion. To testify this hypothesis, an additional Th1 ATAC-seq result (GSE# 137383) was introduced to the analysis (22). In consistent with the similarity between pathogenic Th17 and pro-inflammatory Th1 in pathogenic features, we found that the accessibility to concordant patho-ChARs, rather than concordant reg-ChARs, was preferred in Th1 (**Figure 2E**). In contrast, the accessibility to unconcordant patho-ChARs and reg-ChARs was almost comparable in Th1, suggesting that these ChARs were irrelevant with the Th17 to Th1 conversion (**Figure 2F**). Therefore, the concordant patho-ChARs and reg-ChARs probably closely associated with the Th17 to Th1 conversion, which were critical in the pathogenicity of Th17. In other words, the dynamic change of accessibility to these concordant ChARs, to some extent, could reflect the status of T cells during the Th17 to Th1 conversion.

### Concordant Patho-ChARs Related Gene Loci Are Sequentially Accessible From Proximal Region to Distal Region During Th17 to Th1 Conversion

**Figure 3:**
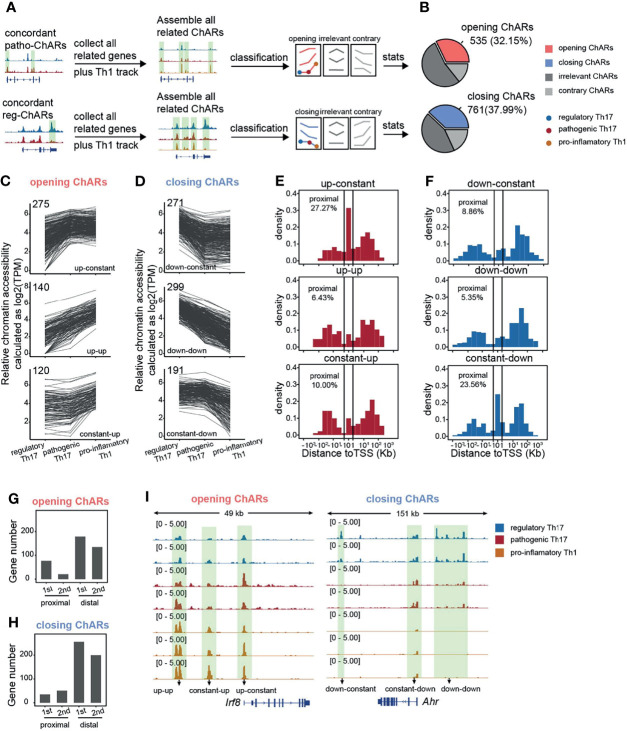
Gene loci related to concordant patho-ChARs are sequentially opened from TSS-proximal region to TSS-distal region during Th17 to Th1 conversion. **(A)** Workflow of identifying all ChARs associated with patho-ChAR or reg-ChARs related genes. **(B)** Statistics of opening ChARs and closing ChARs during the regulatory Th17 to pathogenic Th17 conversion. Opening ChARs and closing ChARs, display concordant accessibility changes (fold change >1.5) in at least one step of the conversion, and never show any contrary changes; contrary ChARs, display contrary accessibility changes (fold change >1.5) in at least one step of the conversion; irrelevant ChARs, the residual ChARs. **(C)** Line plots of opening ChARs with ‘up-constant’, ‘up-up’, and ‘constant-up’ modes. **(D)** Line plots of closing ChARs with ‘down-constant’, ‘down-down’, and ‘constant-down’ modes. **(E)** Distance of opening-ChARs with ‘up-constant’, ‘up-up’, ‘constant-up’ modes to their neighboring TSSs, respectively. Percentage of ChARs found within +1 to -1 Kb of TSS were calculated. **(F)** Distance of closing-ChARs with ‘down-constant’, ‘down-down’, and ‘constant-down’ modes to their neighboring TSSs, respectively. Percentage of ChARs found within +1 to -1 Kb of TSS were calculated. **(G)** Counting for the numbers of genes related to opening-ChAR changes in TSS-proximal or TSS-distal regions in two steps of the Th17 to Th1 conversion. 1^st^, regulatory Th17 to pathogenic Th17 conversion; 2^nd^, pathogenic Th17 to pro-inflammatory Th1 conversion. **(H)** Counting for the numbers of genes related to closing-ChAR changes in TSS-proximal or TSS-distal regions in two steps of the Th17 to Th1 conversion. 1^st^, regulatory Th17 to pathogenic Th17 conversion; 2^nd^, pathogenic Th17 to pro-inflammatory Th1 conversion. **(I)** ATAC-Seq tracks at *Irf8* (particular opening-ChAR related gene) and *Ahr* (particular closing-ChAR related gene) loci, with differential peaks highlighted in green.

Next, we further explored, during the Th17 to Th1 conversion, how chromatin landscape change happened with genes related to the concordant patho-ChARs or reg-ChARs. We collected concordant patho-ChARs and reg-ChARs related genes, and profiled all their ChARs in regulatory Th17, pathogenic Th17, as well as pro-inflammatory Th1 (**Figure 3A**). Concordant patho-ChARs were just found to correlate with the Th17 to Th1 conversion (**Figure 2E**). So, here, we further screened these ChARs with a scenario that they should display concordant accessibility changes in at least one step of the conversion, and they should never show any contrary changes. Accordingly, the ChARs for patho-ChARs and reg-ChARs related genes were divided into three subsets, concordant ChARs, contrary ChARs, and irrelevant ChARs (**Figure 3B**). The concordant ChARs were isolated for further analyses. Within them, those associated with patho-ChAR-related genes were termed as ‘opening’ ChARs, while the residuals associated with reg-ChARs related genes were termed ‘closing’ ChARs. The proportion of them reached to about 30~40%, suggesting that they might play essential roles in regulating the expression of their related genes (**Figure 3B**). Then, the opening and closing ChARs were further classified based on their changes in the Th17 to Th1 conversion. As a result, the ‘opening’ ChARs were classified into ‘up-constant’, ‘up-up’, and ‘constant-up’ clusters (**Figure 3C**), while the ‘closing’ ChARs were classified into ‘down-constant’, ‘down-down’, and ‘constant-down’ clusters (**Figure 3D**). Accessibility to promoter in TSS-proximal region was always required for initiating a gene expression, whereas accessibility to enhancers mostly in TSS-distal region was not usually necessary (18). So, according to gene expression change during the Th17 to Th1 switch, we wondered how their chromatin accessibility in TSS-proximal and TSS-distal regions was changed. Thus, the distribution of ‘opening’ and ‘closing’ ChARs in their gene loci were analyzed. Intriguingly, only in the ‘up-constant’ cluster of ‘opening’ ChARs and the ‘constant-down’ cluster of ‘closing’ ChARs we found an obvious enrichment in TSS-proximal region (**Figures 3E, F**). Given the role of TSS-proximal region in initiating gene expression (19, 23), these results indicated that most TSS-proximal regions of patho-ChARs related genes became accessible in the step of regulatory Th17 to pathogenic Th17 switch. However, for the reg-ChARs related genes, though a large proportion of ChARs in the ‘constant-down’ cluster located in TSS-proximal region, the total number of them in this cluster was relatively low. Hence, it was still hard to say in which step majority of their TSS-proximal ChARs were closed. So, to better describe the temporal and spatial changes of chromatin accessibility during the Th17 to Th1 conversion, we calculated the absolute number of genes associated with the changes in TSS-proximal and TSS-distal regions. Briefly, for genes related to patho-ChARs, their expression concordant TSS-proximal regions became accessible mainly at the regulatory Th17 to pathogenic Th17 step. While, in the pathogenic Th17 to pro-inflammatory Th1 step, the increased chromatin accessibility was mainly observed in expression concordant TSS-distal regions (**Figure 3G**). Thus, during the Th17 to Th1 switch, expression concordant ChARs of patho-ChARs related genes were sequentially opened from TSS-proximal region to TSS-distal region. On the other hand, for genes related to reg-ChARs, the closing of their expression concordant regions was found to be similar in both steps of the switch, reflecting a fact that closing either TSS-proximal ChARs or TSS-distal ChARs could potentially attenuate gene expression (**Figure 3H**). These temporal and spatial changes of chromatin accessibility during the Th17 to Th1 conversion were also confirmed by particular patho-ChARs or reg-ChARs related genes, *Irf8* and *Ahr* (24, 38) (**Figure 3I**). Therefore, this sequential change of opening ChARs might play crucial regulatory roles during the Th17 to Th1 conversion.

### Transcription Factor Binding Motifs Enriched From ‘Opening’ and ‘Closing’ ChARs Exhibit Distinct Chromatin Distribution Patterns Around Gene Loci

**Figure 4:**
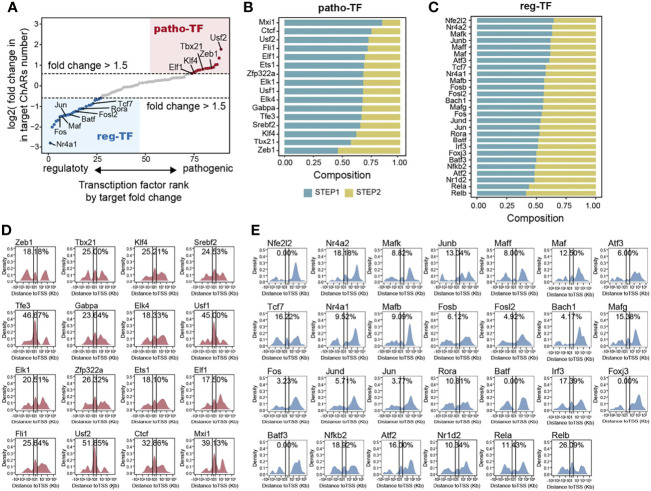
Transcription factor binding motifs enriched from opening- and closing- ChARs exhibit distinct chromatin distribution patterns. **(A)** Transcription factors (TFs) ranked by the ratio of their binding motifs in patho-ChARs to that in reg-ChARs. Transcription factors with more than 1.5fold change (motifs in patho-ChARs/motifs in reg-ChARs) were denoted as patho-TFs, while transcription factors with less than 0.67 fold change (motifs in patho-ChARs/motifs in reg-ChARs) were denoted as reg-TFs. **(B)** Distribution of patho-TFs ranked by the ratio of their binding motifs with increased accessibility in first step of the Th17 to Th1 conversion to those in the second step. **(C)** Distribution of reg-TFs ranked by the ratio of their binding motifs with decreased accessibility in first step of the Th17 to Th1 conversion to those in the second step. **(D)** Distance of patho-TFs targeted binding motifs in opening-ChARs to their neighboring TSSs. Percentage of ChARs found within +1 to -1 Kb of TSS were calculated. **(E)** Distance of reg-TFs targeted binding motifs in closing-ChARs to their neighboring TSSs. Percentage of ChARs found within +1 to -1 Kb of TSS were calculated.

Chromatin accessible regions were permissive for transcription factor binding (11). Therefore, for a particular transcription factor, the frequency of its binding motifs in ChARs could reflect its transcription regulation activity. Thus, we could use it to predict transcriptional regulations during the Th17 to Th1 conversion. First, we calculated binding motifs of all transcription factors in the ‘opening’ ChARs and ‘closing’ ChARs, respectively. Transcription factors with more than 20 overall binding motifs in the two ChARs were then isolated and ranked based on the frequency of their binding motifs in the ‘opening’ and ‘closing’ ChARs (**Figure 4A**). Transcription factors with more than 1.5-fold increase of binding motifs in ‘opening’ ChARs were termed as patho-TFs, whose transcriptional regulation activities tended to be enhanced in pathogenic Th17. Similarly, transcription factors with more than 1.5-fold increase of binding motifs in ‘closing’ ChARs were termed as reg-TFs. According to the literature, many of these patho-TFs and reg-TFs were previously reported to involve in regulating the Th17 to Th1 switch, corroborating the accuracy of our analysis (25–29, 37, 44, 56–76) (**Table S1**).

As we had just found, in the two steps of Th17 to Th1 conversion, TSS-proximal and TSS-distal regions of the patho-ChARs related genes sequentially became accessible. Thus, we wondered whether these predicted patho-TFs also exhibited a temporal and spatial difference in their transcription regulation activities. We ranked patho-TFs based on the proportion of their binding motifs appeared in the first step, which was supposed to correlated with the initiation of their regulated genes (**Figure 4B**). Thus, patho-TFs on the top of the rank were supposed to execute their activities earlier. Next, we examined the distribution of patho-TFs related binding motifs. As we expected, for patho-TFs ranked on the top, such as *Usf2*, *Ctcf*, and *Mxi1*, their binding motifs showed an obvious distribution pattern to TSS-proximal region. Whereas, for patho-TFs ranked at the bottom, such as *Tbx21*, *Zeb1*, and *Klf4*, their binding motifs preferred to allocate in TSS-distal region (**Figure 4D**). Accordingly, these patho-TFs exhibited potentials to orchestrate the sequential change of chromatin accessibility during the Th17 to Th1 switch. On the other hand, reg-TFs were also ranked (**Figure 4C**), and the distributions of their binding motifs were profiled (**Figure 4E**). Binding motifs of reg-TFs preferentially distributed in TSS-distal regions, indicating that they were critical in execute the regulations there. Together, the distribution of these motifs suggested that patho-TFs and reg-TFs were critical in regulating the Th17 to Th1 conversion.

### Single-Cell Transcriptome Confirms the Patho-TF to Reg-TF Change During Th17 to Th1 Switch

**Figure 5:**
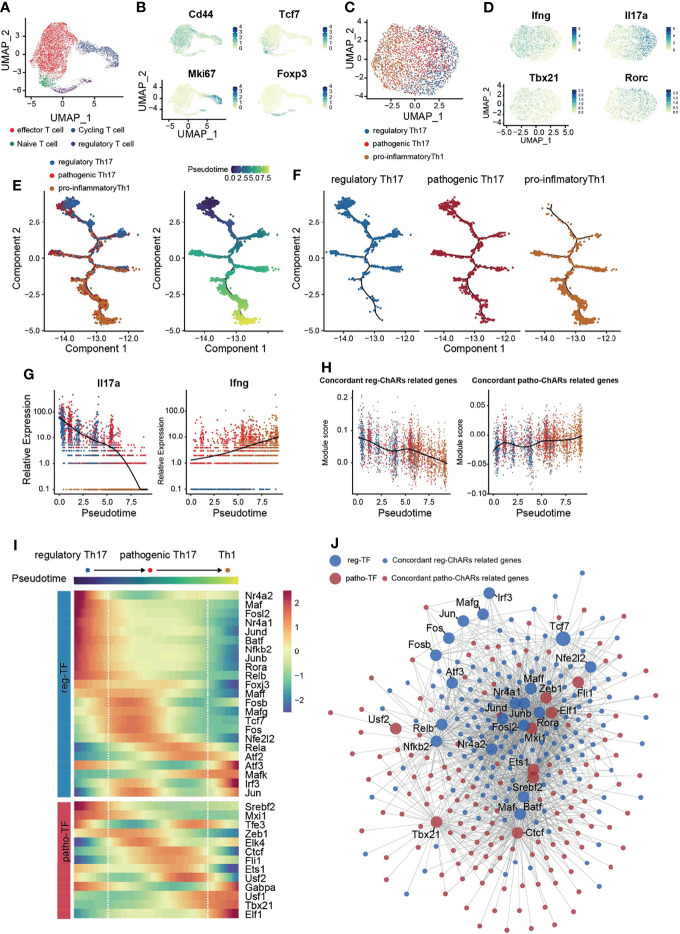
Single-cell transcriptome corroborates a regulatory network change from a reg-TF operated manner to a patho-TF operated manner during the Th17 to Th1 Conversion. **(A)** Single-cell transcriptome (scRNA-Seq) of CD4^+^ T cells in the CNS of EAE mice. **(B)** Expression of *Cd44*, *Tcf7*, *Mki67*, and *Foxp3* in CD4^+^ T cells in the CNS of EAE mice. **(C)** Evaluation of regulatory Th17, pathogenic Th17, and pro-inflammatory Th1 features in cells isolated from the Teff cluster. **(D)** Expression of *Ifng*, *Il17a*, *Tbx21*, and *Rorc* in the isolated cells from the Teff cluster. **(E)** Pseudotime analysis of the isolated regulatory Th17, pathogenic Th17, and pro-inflammatory Th1 cells. Each dot represents an individual cell, colored by cluster (left) or by pseudotime(right). **(F)** Distribution of regulatory Th17, pathogenic Th17, and pro-inflammatory Th1 cells along the pseudotime trajectory. **(G)** Dynamic expression change of *Ifng* and *Il17a* along the pseudotime trajectory. **(H)** Dynamic change of patho-ChARs and reg-ChARs related gene features along the pseudotime trajectory. **(I)** Heatmap of patho-TFs and reg-TFs along the pseudotime trajectory. **(J)** Transcriptional regulatory network constructed by patho-TFs and reg-TFs and their targets from patho-ChARs and reg- ChARs related genes.

Next, to confirm the regulatory role of patho-TFs and reg-TFs during Th17 to Th1 switch, we performed a single-cell RNA-sequencing analysis of CNS T cells from EAE mice (GSE188161) (**Figure 5A**). Four T cells clusters were identified based on their signature gene expression, effector T (Teff), naïve/memory T (Tn/m), regulatory T (Treg), and proliferating T (Tcyc) (**Figure 5B**). We then collected Th1/Th17 cells from the Teff cluster, based on their expression of *Rorc*, *Tbx21*, *Il17a*, and *Ifng*, and further classified them into regulatory Th17, pathogenic Th17, and pro-inflammatory Th1 subgroups (**Figures 5C, D**). As expected, pseudotime analysis on these Th1/Th17 cells displayed a conversion trajectory from the regulatory Th17 to pro-inflammatory Th1 (**Figures 5E, F**). Along the trajectory, *Il17a* expression was gradually reduced, while *Ifng* expression was increased, corroborating that it was consistent to the Th17 to Th1 conversion (**Figure 5G**). Moreover, patho-ChARs related gene feature also increased along the trajectory, whereas reg-ChARs related gene feature was attenuated (**Figure 5H**). Then, we further assessed the expression of patho-TFs and reg-TFs. While most pro-inflammatory Th1 related TFs, such as *Tbx21*, *Elf1*, and *Usf1*, exhibited a gradually increased expression, majority of the patho-TFs also reached their highest expression before converting to Th1. Whereas, some patho-TFs showed culminating expression level even at the beginning of the trajectory, like *Srebf2* and *Mxi1* (**Figure 5I**). In contrast, the expression of reg-TFs was mostly reduced at the regulatory Th17 to pathogenic Th17 step. Nevertheless, a few of them were also found to highly express at the Th1 stage, indicating that they probably executed transcriptional suppressive roles (**Figure 5I**). Thus, these results corroborated that patho-TFs and reg-TFs were sequentially activated along the regulatory Th17 to pro-inflammatory Th1 trajectory. To examine the transcriptional regulation change during Th17 to Th1 conversion, we also constructed a regulatory network with the pahto-TFs and reg-TFs and their regulated genes, according to their expression correlation from the scRNA-seq result. Clearly, we did find that patho-TFs and reg-TFs mainly correlated with their related genes (**Figure 5J**). Together with the gene expression changes, these results also suggested that the regulatory network was sequentially changed during the Th17 to Th1 conversion.

## Discussion

**Figure 6:**
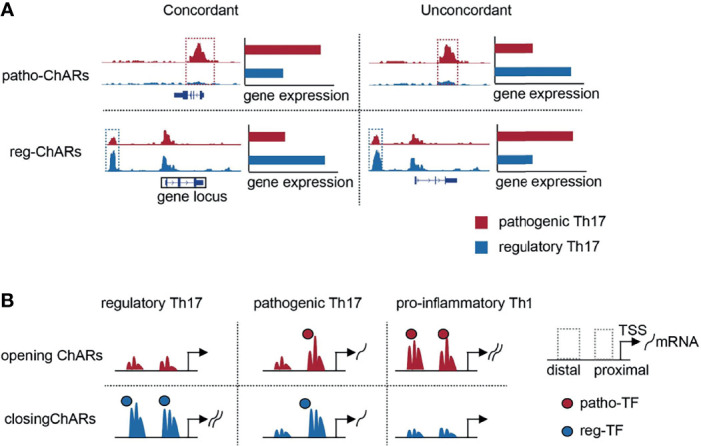
Schematic overview of the study. **(A)** Schematic diagrams of patho-ChARs, reg-ChARs, concordant ChARs, unconcordant ChARs and gene locus. **(B)** Dynamic changes of opening ChARs and closing in the process of Th17 to Th1 conversion.

Pathogenic Th17 are involved in the occurrence and development of many autoimmune diseases. Unravelling the underlying mechanisms associated with their generation and further differentiation to pro-inflammatory Th1is important clinically. Thus, difference between pathogenic Th17 and the other conventional regulatory Th17 has been comprehensively compared in the past (77). Most of these studies were focused on gene expression, to discover specific regulators in the pathogenic Th17 and illuminate their roles in autoimmune responses. These comparisons were also processed at single-cell level recently, which further revealed the dynamics of gene expression change during the regulatory Th17 to pathogenic Th17 switch (32). Gene expression change is closely associated with alterations in chromatin (11, 12). Accessible regions in chromatin contain regulatory elements, such as transcription factor binding motifs (11, 12). Therefore, discovering the chromatin accessibility change will provide additional information about the regulatory mechanisms underlying pathogenic Th17 generation. However, such studies are still relatively rare now. Here, the expression concordant ChARs are particularly analyzed. These ChARs are rich in promoters and enhancers, and are particularly important in regulating gene expression. We have identified a cluster of ‘opening’ ChARs in the pathogenic Th17, and revealed that the access to them exhibited a significant temporal and spatial difference during the Th17 to Th1 conversion (**Figure 6**).

Within the ‘opening’ ChARs, the ones located in TSS-proximal region contain promoters, whose accessibilities are prerequisites for gene expression (11, 12). We found that their accessibilities are mostly settled in the regulatory Th17 to pathogenic Th17 change. This opening of these TSS-proximal regions meets the essential requirements for initiating the expression of these pro-inflammatory genes. Interestingly, com-ChARs were also found to mainly locate in TSS-proximal regions, indicating that the regulation of these genes should be relatively concise. Since the gene expression regulation is energy consuming, this proximal regulation probably is the most economical way for the expression of most genes in a cell. Similarly, the proximal regulation of the pro-inflammatory genes in pathogenic Th17 can ensure an efficient expression of them. But, in another perspective, their expression level probably cannot be properly regulated, which may eventually result in the occurrence of inflammatory responses. In contrast, the effector genes in regulatory Th17 are obviously regulated in a distinct way. Their reg-ChARs are predominantly distributed in TSS-distal regions. Moreover, in either proximal or distal regions, there are also many ChARs irrelevant or contrary with the gene expression. These observations suggest that the regulation of these immune effector genes is more complicated, and negative regulatory mechanisms perhaps prevalently exist to avoid an unleashed immune response. Therefore, the shortage of distal ChARs, especially those with negative regulatory roles, probably correlates with the pathogenicity of Th17.

During the next pathogenic Th17 to pro-inflammatory Th1 switch, we have found that increased access to the expression concordant ‘opening’ ChARs mainly occurs in TSS-distal region, indicating that these additional regulations finally lead to the switch to Th1. Related with our finding, a previous study also reported that, at *Ifng* locus, diverse distal regulatory elements are required at distinct stages of Th1 differentiation (23, 78). Therefore, after establishing the proximal and basic distal regulations for those pro-inflammatory genes in the pathogenic Th17, the residual ‘opening’ ChARs in TSS-distal regions become more critical in deciding the conversion to Th1. TSS-distal regions are more complicated than TSS-proximal regions, containing both positive and negative regulatory elements (11, 12). In our study, to simplify the analyses, we only focused on the expression concordant ChARs. But we did find that a substantial proportion of ChARs were contrary to the expression of ‘opening’ ChAR-related genes. They may serve as suppressors for these pro-inflammatory genes, and thus are supposed to be useful for protecting the pathogenicity of Th17. Therefore, a further study of them may help to find new ways to ameliorate the pro-inflammatory response by Th17 or Th1.

Distinct with the sequential access to opening ChARs, the attenuation of closing ChARs during the Th17 to Th1 switch is not particularly arranged. In the two steps of Th17 to Th1 conversion, equivalent numbers of TSS-proximal and TSS-distal closing ChARs are turned off, consistent with the fact that destructing either proximal or distal regulations could disrupt gene expression. Together with the change of opening ChARs, it suggests that the establishment of gene expression should be precisely organized. It may also be of interests to explore the dynamic accessibility change of these closing ChARs during the naïve CD4^+^ T to regulatory Th17 differentiation. Moreover, our observations also indicate that, to attenuate the conversion of Th17 to Th1, these opening ChARs could be equally considered, no matter in which steps of the conversion they become accessible. However, according the fact that pathogenic Th17 themselves can execute severe inflammatory responses (54), the opening ChARs changed at the earlier step of Th17 to Th1 conversion should be considered with high priority.

To further elucidate the regulatory mechanism during the Th17 to Th1 conversion, transcription factors involved in the regulation of opening ChAR-related genes have also been investigated. These transcription factors are predicted according to the appearance of their binding motifs in opening ChARs. Majority of them have been previously reported to participate in regulating the pathogenic Th17 (44, 68–76). Thus, these known transcription factors, as well as the residual ones we identified in this study, are all worth further exploring. The frequency of their binding motifs in opening ChARs suggests that their regulatory roles in the Th17 to Th1 switch are distinct. Transcription factors such as *Usf2*, *Ctcf*, and *Fli1* have displayed a significant binding preference to TSS-proximal region, indicating that they are more critical in initiating gene expression at the regulatory Th17 to pathogenic Th17 step. Whereas other transcription factors preferentially binding to TSS-distal region may boost the pathogenic Th17 to Th1 switch. This functional differences of these transcription factors were also confirmed by the single-cell analysis in our study. Our results have also provided a scenario to evaluate the role of different regulators in driving the pathogenicity of Th17. Transcriptional regulators with more TSS-proximal binding sites in the ‘opening’ ChARs probably need more attentions in future.

The pathogenicity of Th17 and their final conversion to pro-inflammatory Th1 are found in many autoimmune diseases (53, 54). Thus, our study reveals a novel regulatory mechanism during this process, which will provide new therapeutic strategies for these diseases. Even though it is difficult to directly change the chromatin accessibility, it could still be indirectly operated *via* transcriptional or epigenetic regulators (11, 12). These operations will require a further understanding of the relationship between chromatin accessibility and other regulations. Nevertheless, our results provide another way to consider the therapy of these autoimmune diseases.

## Data Availability Statement

The datasets presented in this study can be found in online repositories. The names of the repository/repositories and accession number(s) can be found in the article/**Supplementary Materials**.

## Author Contributions

LH and CZ conceived the project. LH performed the majority of the bioinformatic analysis. CZ and BL contributed to result discussion and analysis. XZ, PL, YYZ, YMZ, YS, YW, and XS helped with some analysis and discussion. LH and CZ wrote the manuscript. CZ supervised the project. All authors contributed to the article and approved the submitted version.

## Funding

This study was supported by the National Natural Science Foundation of China (32170896, 31770957 and 91842102) and the Natural Science Foundation of Beijing (18G10645) to CZ.

## Conflict of Interest

The authors declare that the research was conducted in the absence of any commercial or financial relationships that could be construed as a potential conflict of interest.

## Publisher’s Note

All claims expressed in this article are solely those of the authors and do not necessarily represent those of their affiliated organizations, or those of the publisher, the editors and the reviewers. Any product that may be evaluated in this article, or claim that may be made by its manufacturer, is not guaranteed or endorsed by the publisher.
