# Supplementary material for: Proximal and Distal Regions of Pathogenic Th17 Related Chromatin Loci Are Sequentially Accessible During Pathogenicity of Th17
Source: Front Immunol. 2022 Apr 19;13:864314. doi: 10.3389/fimmu.2022.864314 (PMC9062102; doi:10.3389/fimmu.2022.864314)
Supplement: Supplementary file 1 [file DataSheet_1.pdf]

SUPPLEMENTARY MATERIAL

Figure S1

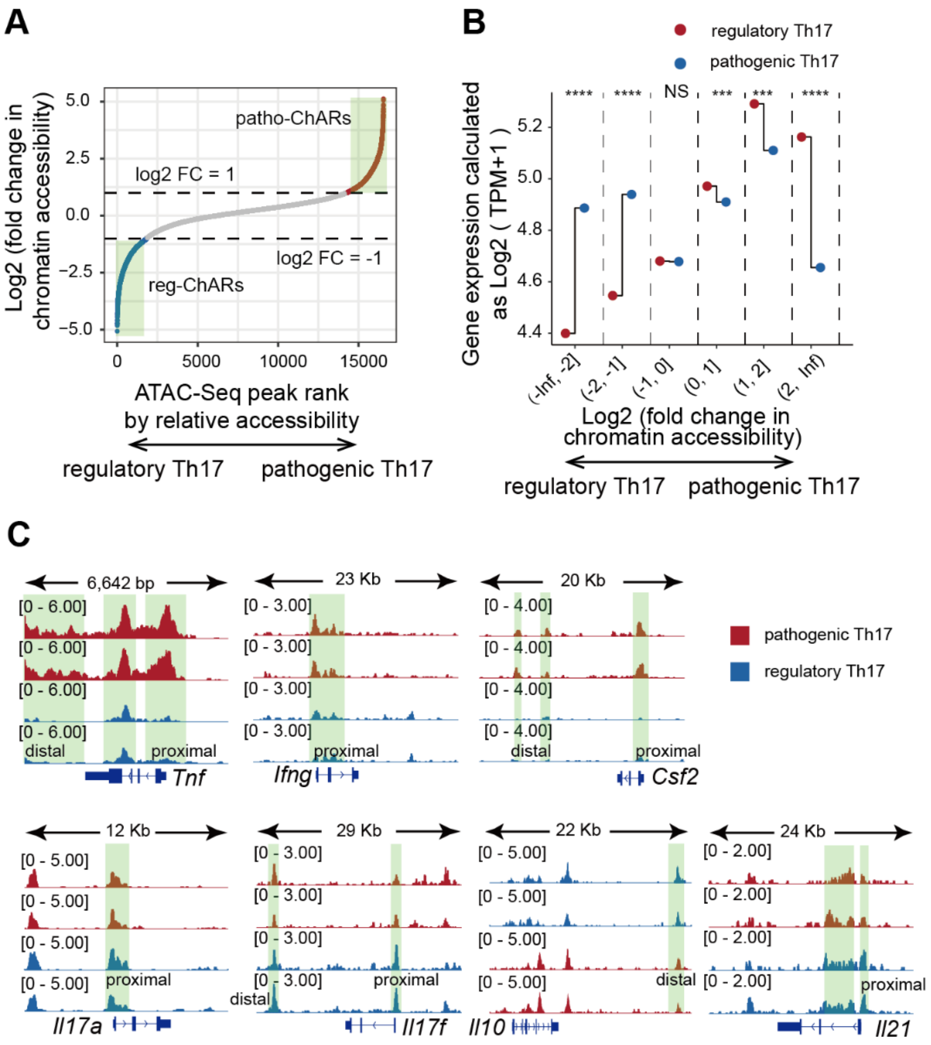

Figure S1. DNA accessibility in pathogenic and regulatory Th17 correlates with the expression of their related genes. **(A)** Distribution of ATAC-Seq peaks ranked by relative chromatin accessibility. Red, patho-ChARs, blue, reg-ChARs, (fold change > 2). **(B)** Average correlation between ChAR accessibility and the expression their neighboring genes in pathogenic and regulatory Th17. ***P<0.001 and ****P<0.0001**.** **(C)** ATAC-Seq tracks at the pathogenic-specific (*Tnf*, *Ifng, Il10* and *Csf2*) and regulatory-specific (*Il17a*, *Il17f* and *Il21*) gene loci, with differential peaks highlighted in green. proximal: TSS-proximal regions; distal: TSS-distal regions.

Figure S2

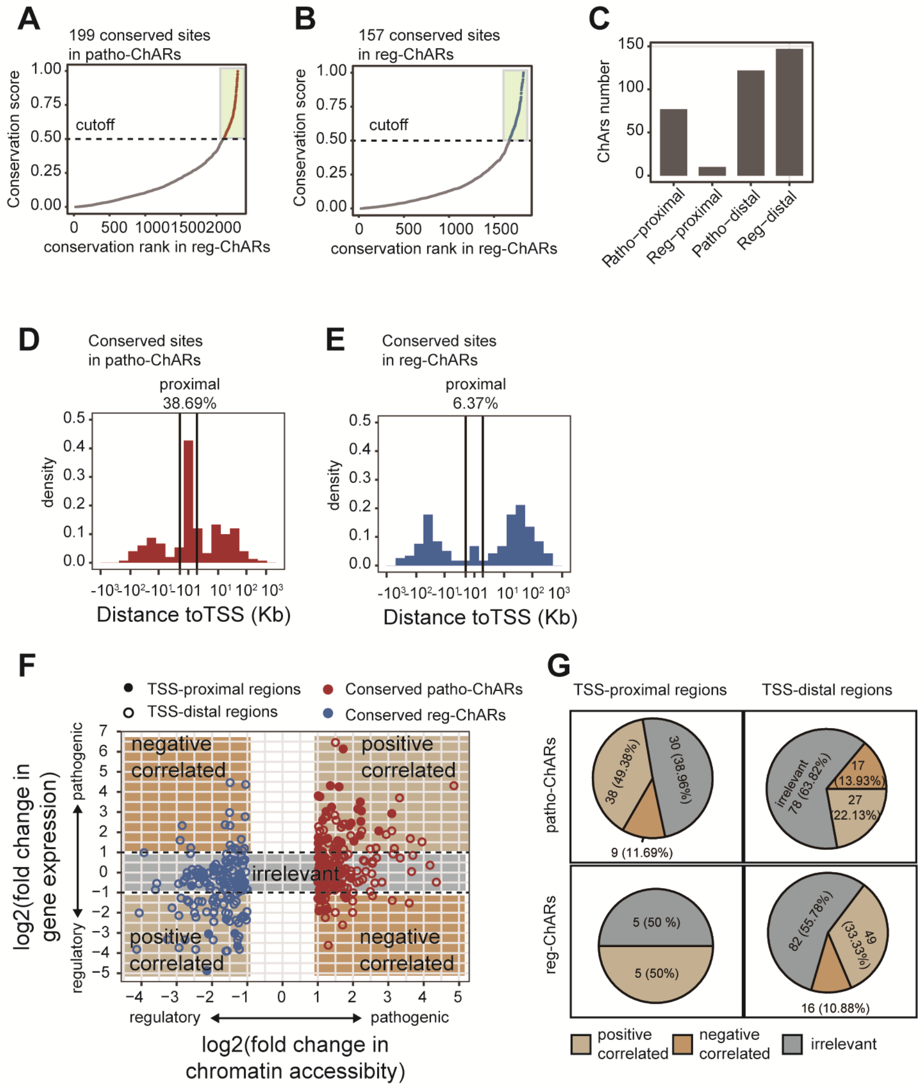

Figure S2. Conserved patho- and reg- ChARs display similar distribution patterns. **(A)** Conservation elbow plot of patho-ChARs. Red points denote the conserved sites, conservation score > 0.5. **(B)** Conservation elbow plot of reg-ChARs. Blue points denote the conserved sites, conservation score > 0.5. **(C)** Statistics of conserved patho-ChARs and conserved reg-ChARs in TSS-proximal and TSS-distal regions. **(D)** Distance of conserved patho-ChARs to their neighboring TSSs. Percent of ChARs found within +1 to -1 Kb of TSS were calculated. **(E)** Distance of conserved reg-ChARs to their neighboring TSSs. Percent of ChARs found within +1 to -1 Kb of TSS were calculated. **(F)** Correlation between the accessibility of conserved ChARs at TSS-proximal or TSS-distal region and the expression of their related genes. **(G)** Statistics of the correlation between patho- and reg-ChARs located at TSS-proximal or TSS-distal regions and the expression of their neighboring genes. Positively correlated ChARs, fold change > 2, negatively correlated ChARs, fold change < 0.5, expression-irrelevant ChARs, 0.5 < fold change < 2.

Table S1

Reg-TF	Reference
Nfe2l2	Kim Ohl, et al. Nuclear Factor Erythroid 2-related Factor 2 Deficiency Exacerbates Lupus Nephritis in B6/lpr mice by Regulating Th17 Cell Function
Nr4a2	Ben J. E. Raveney, et al. Nuclear receptor NR4A2 orchestrates Th17 cell-mediated autoimmune inflammation via IL-21 signalling
Mafk	not reported
Junb	Tiffany M. Carr, et al. JunB promotes Th17 cell identity and restrains alternative CD4+ T-cell programs during inflammation
Maff	not reported
Maf	Kojiro Sato, et al. Marked induction of c-Maf protein during Th17 cell differentiation and its implication in memory Th cell development
Atf3	Doaa Glal, et al. ATF3 Sustains IL-22-Induced STAT3 Phosphorylation to Maintain Mucosal Immunity Through Inhibiting Phosphatases
Nr4a1	Li-Mei Wang, et al. Nr4a1 plays a crucial modulatory role in Th1/Th17 cell responses and CNS autoimmunity
Tcf7	Pawel Muranski, et al. Th17 cells are long-lived and retain a stem cell-like molecular signature
Mafb	Dhouha Daassi, et al. Differential expression patterns of MafB and c-Maf in macrophages in vivo and in vitro
Fosb	Jinfang Zhu, et al. Differentiation of Effector CD4 T Cell Populations*
Fosl2	Shetty, A, et al. Interactome Networks of FOSL1 and FOSL2 in Human Th17 Cells
Bach1	Kim, S. K, et al. The novel Bach1 inhibitor HPP971 uniquely activates Nrf2 and reduces disease severity in a mouse model of experimental autoimmune encephalomyelitis
Mafg	not reported
Fos	Jinfang Zhu, et al. Differentiation of Effector CD4 T Cell Populations*
Jund	Jinfang Zhu, et al. Differentiation of Effector CD4 T Cell Populations*
Jun	Jinfang Zhu, et al. Differentiation of Effector CD4 T Cell Populations*
Rora	June-Yong Lee, et al. RORa enforces stability of the T-helper-17 cell effector program
Batf	Schraml BU, et al. The AP-1 transcription factor Batf controls T(H)17 differentiation
Irf3	Denise C Fitzgerald, et al. Interferon regulatory factor (IRF) 3 is critical for the development of experimental autoimmune encephalomyelitis
Foxj3	not reported
Batf3	Wonyong Lee, et al. The transcription factor Batf3 inhibits the differentiation of regulatory T cells in the periphery
Nfkb2	Wai Po Chong, et al. The Cytokine IL-17A Limits Th17 Pathogenicity via a Negative Feedback Loop Driven by Autocrine Induction of IL-24
Atf2	not reported
Nr1d2	not reported
Rela	Xuebin Qu, et al. TLR4-RelA-miR-30a signal pathway regulates Th17 differentiation during experimental autoimmune encephalomyelitis development
Relb	Ievgen O. Koliesnik, et al. RelB regulates Th17 differentiation in a cell-intrinsic manner

Patho-TF	Reference
Mxi1	Jing Chen, et al. Interaction of RNA-binding protein HuR and miR-466i regulates GM-CSF expression
Ctcf	Yu, Xiaofei et al. TH17 cell differentiation is regulated by the circadian clock
Usf2	Dan Hu, et al. Aberrant expression of USF2 in refractory rheumatoid arthritis and its regulation of proinflammatory cytokines in Th17 cells
Fli1	Miura S, et al. Fli1 deficiency suppresses RALDH1 activity of dermal dendritic cells and related induction of regulatory T cells: a possible role in scleroderma

Elf1	not reported
Zfp322a	not reported
Elk1	not reported
Usf1	Ratajewski M, et al. Upstream stimulating factors regulate the expression of RORγT in human lymphocytes
Elk4	Ping Hsien Lee, et al. The Transcription Factor E74-Like Factor 4 Suppresses Differentiation of Proliferating CD4+ T Cells to the Th17 Lineage.
Gabpa	not reported
Tfe3	not reported
Srebf2	Guoliang Cui, et al. Liver X receptor (LXR) mediates negative regulation of mouse and human Th17 differentiation
Klf4	Lori Lebson, et al. Cutting edge: The transcription factor Kruppel-like factor 4 regulates the differentiation of Th17 cells independently of RORγt
Tbx21	Yuhong, et al. T-bet is essential for encephalitogenicity of both Th1 and Th17 cells
Zeb1	Qian Y, et al. ZEB1 promotes pathogenic Th1 and Th17 cell differentiation in multiple sclerosis
